# Surface Physical and Mechanical Properties of Short Fibre Reinforced Composite Resins in Direct Restorative Dentistry: A Systematic Review

**DOI:** 10.1111/adj.70051

**Published:** 2026-05-11

**Authors:** Murray Macpherson, Michael Liddell, Touraj Nejatian

**Affiliations:** ^1^ College of Medicine and Dentistry James Cook University Cairns Queensland Australia; ^2^ College of Science and Engineering James Cook University Cairns Queensland Australia

**Keywords:** direct dental restoration, short fibre reinforced composite resin, surface properties

## Abstract

**Background:**

Short Fibre Reinforced Composite resins may be used to restore odontogenic tissues and have enhanced physical and mechanical properties compared with conventional resins. However, their surface characteristics remain a concern, and this has limited their use as direct dental restorative materials.

**Methods:**

Five published and three grey literature electronic databases were searched in accordance with Preferred Reporting Items for Systematic Reviews and Meta‐Analyses guidelines.

**Results:**

This systematic review identified and assessed studies that examined surface hardness, surface roughness, surface wear, water solubility, water sorption, and surface energy of SFRC resins. Most studies were in vitro (*n* = 30), and only two clinical trials were identified. Minimal grey literature was found, all originating from manufacturers. The evidence from these studies suggests that matrix composition and fibre characteristics influence surface properties, consistent with findings reported for conventional particle filled composite resins.

**Conclusions:**

The physical and mechanical surface properties of SFRC resins used as direct dental restorative materials appear appropriate and stable in the oral environment; however, long‐term clinical studies are needed to confirm this.

## Clinical Relevance

Short fibre reinforced composite (SFRC) resin has been developed primarily as a restorative material to exhibit physical and mechanical properties similar to those of dental tissues being replaced, some of which are designed to be bulk filled. However, concerns about the surface characteristics of SFRC resin have constrained widespread adoption as a dental restorative material. This review synthesises the current evidence on surface properties of exposed SFRC resin to inform its appropriate clinical application.

## 1 Introduction

Loss of odontogenic tissues can arise from carious or non‐carious processes [1]. Natural teeth possess complex characteristics that can be described by the physical, chemical, mechanical, and optical properties of their constituent tissues [2]. A wide variety of restorative materials, such as metals, ceramics, and composite resins, are currently used to replace lost odontogenic tissues [2]. The development of odontogenic restorative materials intended to replicate and replace the lost odontogenic tissues is therefore important for the longevity and effectiveness of restorative treatments [2].

Two key aspects to the long‐term clinical success of restorative dental materials are (i) the prevention of further damage to the restored tooth and adjacent teeth, and (ii) the surface characteristics of the restorative material used [1, 2, 3].

Dental restorations can be categorised as (i) direct restorations, which are constructed intraorally, and (ii) indirect, which are fabricated extraorally [1]. Overall, the physical and mechanical surface properties of indirect restorative materials tend to be superior to those of direct materials [1, 2]. Direct restorations are typically more cost‐effective and time‐efficient but demonstrate lower longevity than indirect restorations [1]. Direct restorative materials can be used in conjunction with indirect restorations or for the complete replacement of the missing odontogenic tissue [2]. Composite resins represent one of the most commonly used direct dental materials and consist of a resin matrix, filler particles, coupling agents, and activators and initiators [4]. In addition, short fibres have been incorporated alongside traditional filler particles in commercial composite resins, leading to the development of short fibre reinforced composite (SFRC) resin [5, 6]. The inclusion of short discontinuous fibres has been reported to enhance specific physical and mechanical properties of these composite resins [5, 6]. When the SFRC resins are used as a complete replacement for the missing tissues, concerns have been raised regarding fibre protrusions on the surface of SFRC resins, as these may increase surface wear on the opposing and adjacent teeth, thereby posing a potential clinical risk [5]. Consequently, manufacturers recommend applying a 2 mm layer of particulate filled composite (PFC) resin over SFRC resins to prevent exposure of short fibres at the restorative surface [7, 8]. Despite this, some researchers have suggested SFRC resins exhibit physical and mechanical surface properties that make them adequate for exposed clinical use [9]. As a result, uncertainty has arisen regarding the suitability of SFRC resins for use in an exposed oral environment.

This review synthesises the relevant literature evaluating the physical and mechanical surface properties of SFRC resins used as direct dental restorative materials when used as a complete replacement of the odontogenic tissues to address this uncertainty.

## 2 Materials and Methods

### 2.1 Protocol and Registration

This systematic review was conducted in accordance with the Preferred Reporting Items for Systematic Reviews and Meta‐Analyses (PRISMA) guidelines [10] and was registered with PROSPERO (CRD420251023100).

### 2.2 Eligibility Criteria

The records searched for in this systematic review comprised case reports, in vitro studies, and ex vivo studies. The search strategy combined the use of Medical Subject Headings (MeSH) and keywords (title/abstract) to maximise sensitivity. Inclusion criteria were: (a) dental‐related studies, (b) inclusion of short fibres in direct dental composite resin, (c) investigation of physical and or mechanical surface properties, and (d) full text available in English.

Studies were excluded from the review if: (a) the full text was unavailable, (b) the record was a review article, (c) the study did not examine surface properties, (d) the study did not use cured composite resin as a direct restorative material, or (e) the study did not investigate short fibre reinforced composite resin.

### 2.3 Information Sources

Computerised searches were performed using electronic databases. Published records were retrieved from MEDLINE (Ovid), CINAHL, Web of Science, Scopus, and PubMed, while grey literature was identified via Trove, WorldCat, and Google Search. No restrictions were applied regarding publication date, language, or source type.

### 2.4 Focus Question

The search question was formulated using the PICO (Population, Intervention, Comparison, and Outcome) framework: “Does the inclusion of short fibres in resins affect the physical and mechanical surface properties in composite resins when used for direct dental restorations?”

### 2.5 Search Strategy

The PICO question guided the analyses and informed the refinement of searches in both published and grey literature. PICO question concepts were defined as direct dental restorations (Population), inclusion of short fibres in resin fillers (Intervention), particle‐filled resin only (Comparison), and physical and mechanical surface properties (Outcome).

Digital database searches were conducted using a combination of relevant MeSH terms and keywords to align with the PICO concepts. Each database was searched in May 2025, and searches were optimised by ensuring that: (a) the number of search results was fewer than the total number of articles available in the database; (b) syntax variations for the database were considered; (c) no syntax errors were present; and (d) character limits were not exceeded. The search strategy used terms outlined in Table 1, which were searched as keywords within the title or abstract or as MeSH terms.

**TABLE 1:** Search strategy for database searches.

Data source	Search terms: keywords/subject headings	Database	Search terms: keywords/subject headings
Google search (Search engine)	“short fiber reinforced” OR “nano fiber reinforced” OR “nanofiber reinforced” OR “glass fiber reinforced” OR “short fibre reinforced” OR “nano fibre reinforced” OR “nanofibre reinforced” OR “glass fibre reinforced” AND “composite resin” OR “resin composite” AND “surface proper*” OR “surface roughness” OR “surface microhardness” OR “surface character*” OR “surface abrasion” OR “surface adsorption” OR “surface absorption” OR “surface wear” AND “dentistry” OR “dental” AND “restoration” OR “restorative”	Scopus	(ALL ((“short fiber” OR “short fibre” OR SFRC) AND reinforced) AND ALL ((“restoration” OR “restorative”)) AND ALL ((“surface character*” OR “surface wear” OR “surface adsorption” OR “surface absorption” OR “surface micro hardness” OR “surface prop*” OR “surface hard*” OR “surface rough*”)) AND ALL (dental OR dentistry) AND ALL ((“composite” OR “resin”)))
		PubMed	(((((“short fiber” OR “short fibre” OR SFRC) AND reinforced) AND ((“restoration” OR “restorative”))) AND ((“surface character*” OR “surface wear” OR “surface adsorption” OR “surface absorption” OR “surface micro hardness” OR “surface prop*” OR “surface hard*” OR “surface rough*”))) AND (dental OR dentistry)) AND ((“composite” OR “resin”))
WorldCat (Catalogue)	(((((“short fiber” OR “short fibre” OR SFRC) AND reinforced) AND ((“restoration” OR “restorative”))) AND ((“surface character*” OR “surface wear” OR “surface adsorption” OR “surface absorption” OR “surface micro hardness” OR “surface prop*” OR “surface hard*” OR “surface rough*”))) AND (dental OR dentistry)) AND ((“composite” OR “resin”))	CINHL	1. exp resins, synthetic/ or exp composite resins/ 2. ((“short fib*” or “glass fib*” or “nano fib*”) and reinforced).mp 3. exp dental restoration failure/ or exp dental restoration, permanent/ or exp dental restoration, temporary/ 4. exp dental polishing/ or exp dental stress analysis/ 5. rough*.mp. 6. exp Tooth Wear/ or exp Dental Restoration Wear/ 7. exp physical phenomena/ 8. 4 OR 5 OR 6 9. 1 and 2 10. 2 and 7 and 8
Trove (Catalogue)	short fib* “composite resin” surface properties dental	Medline (Ovid)	1.exp resins, synthetic/ or exp composite resins/ 2.((“short fib*” or “glass fib*” or “nano fib*”) and reinforced).mp 3.exp dental restoration failure/ or exp dental restoration, permanent/ or exp dental restoration, temporary/ 4.exp dental polishing/ or exp dental stress analysis/ 5.rough*.mp. 6.exp Tooth Wear/ or exp Dental Restoration Wear/ 7.exp physical phenomena/ 8. 4 OR 5 OR 6 9.1 and 2 10.2 and 7 and 8

### 2.6 Data Collection Process

An independent assessment of eligibility was conducted, involving title and abstract screening, then full‐text review by three assessors (MM, GR, and RL). A fourth reviewer (TN) resolved discrepancies to reach a consensus.

Risk of bias was assessed using the Quality Assessment for In Vitro Studies (QUIN) tool, outlined in Appendix 1. Articles were evaluated as adequately specified (2), inadequately specified (1), not specified (0), or not applicable (−) [11] by three assessors (MM, GR, and AG), and disagreements were resolved by a fourth reviewer (TN).

## 3 Results

### 3.1 Search Results

**FIGURE 1:**
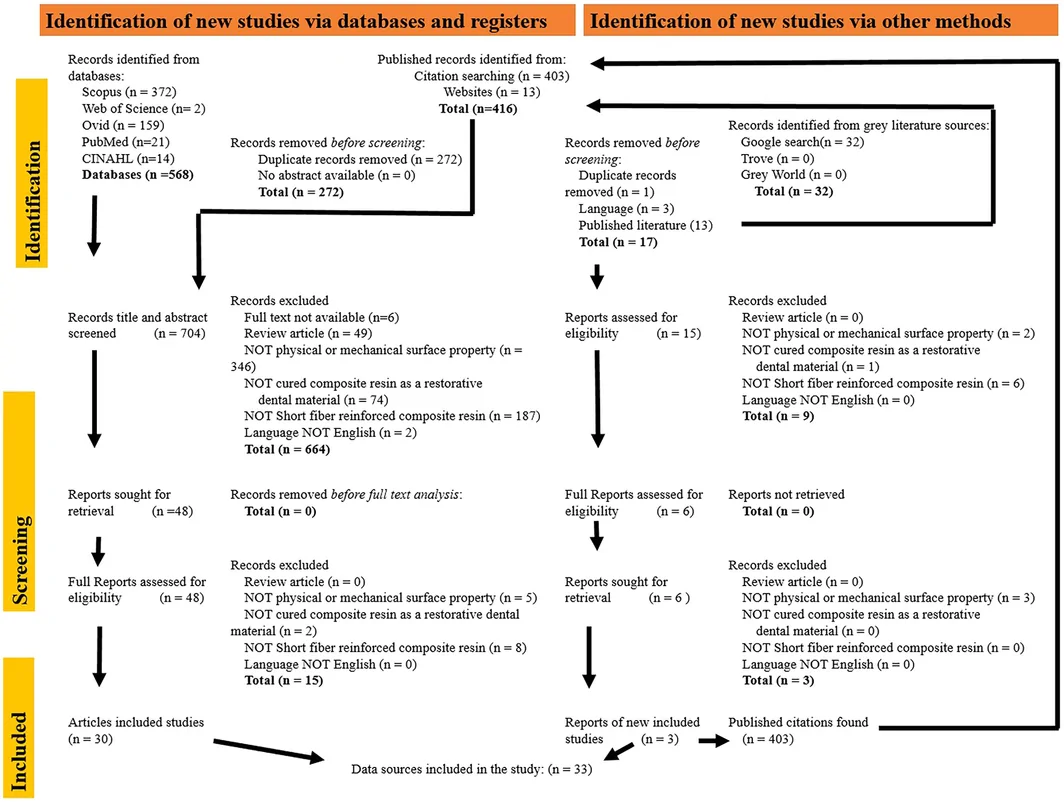
PRISMA diagram.

This systematic review incorporated academic articles (*n* = 30) and grey literature sources (*n* = 3). The results of the searches are shown in the PRISMA diagram (Figure 1). The electronic grey literature searches yielded 32 records, of which 16 were excluded during screening, and 16 were included. Of the 16 included records, 13 were published literature and were added to the published database, and three reports were grey literature and were reviewed accordingly. From the three grey literature reports, 403 references were extracted and added to the published database search.

A total of 984 records were identified in the published literature, 568 from electronic databases, and 416 from grey literature. After removing 272 duplicate records and excluding 679 records, 33 articles remained that were reviewed.

### 3.2 Data Collected

Assessment of 33 records (30 published articles and three grey literature sources) indicated that these studies investigated the physical and mechanical surface properties of SFRC resin, examining aspects of surface hardness, surface roughness, wear, water solubility, water sorption, and surface energy of the restorative surface. The 30 published articles comprised 28 in vitro studies and two clinical trials. All three of the grey literature sources were manufacturers' product information. The extracted data is shown in Appendix 3.

### 3.3 Risk of Bias Tool

In this review, articles were evaluated using the QUIN tool. Final scores were expressed as percentages using the formula below. The articles were classified as low (> 70%), medium (50%–70%), and high (< 50%) risk of bias.

$$\text{Final Score}=\frac{\text{total score}\times 100}{2\times \text{number of criteria applicable}\;}$$

The risk of bias assessment using the QUIN tool found that five (5) articles had a low risk of bias, 25 had a medium risk of bias, and none (0) had a high risk of bias. The product information from the three (3) manufacturers did not present sufficient data to permit an accurate assessment of risk of bias. Results from the risk of bias assessment are displayed in Appendix 2.

#### 3.3.1 Surface Hardness

Surface hardness of SFRC resin was measured by Vickers' surface hardness [12, 13, 14, 15, 16, 17, 18, 19, 20, 21, 22, 23, 24, 25], and nano surface hardness indentation [26, 27]. Comparisons between SFRC resins and PFC resins produced inconsistent results. Several studies reported SFRC resins had an equal or increased hardness relative to PFC resins [12, 13, 14, 25], whereas other studies found a lower hardness for SFRC resins compared with PFC resins [24, 26, 27, 28]. Bulk‐fill SFRC resins exhibit comparable or higher hardness to their PFC bulk‐fill resin counterparts [22, 25], and this increased hardness is associated with improved mechanical properties [23].

Our analysis of the articles found that: (i) water sorption [17] and the presence of Oxygen Inhibition Layer (OIL) [18] contribute to reduced surface hardness in SFRC resins, as has been observed in PFC resins [17, 18], (ii) storage in water, causing water sorption, appears to affect SFRC resins to a lesser extent than PFC resins [16, 24], (iii) hydrothermal ageing [18] and immersion in food‐simulating liquids (FSL) [19] did not significantly affect SFRC resin surface hardness but resulted in significant reduction in PFC resin surface hardness, (iv) exposure of SFRC resins to ethanol softens the composite matrix and consequently decreases surface hardness [17], and (v) microhardness after water storage decreased as curing times increased [24]. This is attributed to the greater plasticizing effect of water on the polymer matrix rather than the rehydration of the silane coupling agent [24].

Increasing curing time has been shown to increase surface hardness of SFRC resin [24]. An increase in nano particle filler weight percent increases surface hardness of SFRC resins; a similar relationship exists with PFC resins [14, 24]. Some evidence suggests that differences in filler composition may influence surface hardness more than curing time [20].

SFRC resin containing 250–350 μm glass fibres maintained a bottom surface hardness greater than 80% of top surface hardness at depths of up to 8 mm, consistent with accepted clinical thresholds for depth of cure [21]. Similar to PFC resin, SFRC resin exhibits reduction in reduces in hardness with increase in depth [20]. However, the magnitude of this reduction is less in SFRC resins than in PFC resins [20, 24].

Bulk placement or layering of SFRC resin in root canal restorations had no significant effect on surface hardness; however, the use of fibre‐reinforced post increased hardness in the coronal and middle regions [27]. In a study examining surface properties of restorative materials after immersion in food‐simulating liquids, SFRC resin showed no significant changes in surface hardness or surface roughness [19].

#### 3.3.2 Wear

Nine (*n* = 10) of the 33 studies investigated the wear resistance of SFRC resin using two‐body wear [18, 25, 28, 29, 30, 31], chewing simulators [32, 33], pin on disc [33, 34], simulated tooth brushing [32], or clinical assessment [9]. Wear can occur as erosion, attrition, and/or abrasion [32]. Contributing factors, such as hydrothermal ageing [18], pH cycling [32], and tooth brushing [32], have also been studied and found to affect the surface wear resistance of SFRC resins. Manufacturers have suggested that SFRC resins exhibit superior wear resistance compared to PFC resins [7, 8]. Silanization of filler and fibre particles in flowable SFRC resins may influence matrix deformation, which could contribute to reduced wear depth [28]. However, packable SFRC resin still provides equivalent or greater wear resistance compared to PFC resins [28, 29, 30, 31, 32].

Fibre plucking is a phenomenon that is described as the loss of protruding fibres from the surface of SFRC resin. Optimising fibre fraction and fibre length may improve the wear characteristics of SFRC resin, potentially reducing fibre plucking [34].

In general, the wear of SFRC resins is less than that of PFC resins [28, 29], and some evidence suggests that fibres wear down with the resin matrix [29]. Although some studies suggest surface roughness may increase after wear due to differential wear between the resin matrix and filler particles [25, 32]. Other studies have suggested additional factors, such as monomer composition and filler types, affect wear resistance [18, 30, 31, 32]. Higher filler volume [18, 33], smaller filler particles [31], Tri‐Ethylene Glycol Di‐Methacrylate (TEGDMA) as a matrix monomer [32], and greater chemical stability of the matrix polymer [30], have been associated with increased wear resistance of SFRC resin.

Critical fibre length is described as the length of fibres that depends on the material and interface properties [30]. Critical fibre length appears to be important for force distribution during wear of the SFRC resins [18, 30, 31, 32, 33]. Fibres that exceed the critical fibre length, reported in some studies to be as much as 50 times the diameter of the fibre, may improve load transfer during weak [30, 31, 32]. In contrast, fibres that are excessively long have been shown to increase surface wear of SFRC resin, suggesting an optimal fibre length [32]. The limited clinical evidence to date suggests that SFRC resin restorations can show satisfactory short‐term wear performance in posterior restorations [9].

Aspect ratio for SFRC resins are a description of the geometric dimensions of the fibres and seeks to describe the lengthiness of fibres [30]. Which has been linked with the structural strength of SFRC resins [30]. The clinical importance of the surface wear depends on the critical fibre aspect ratio, which has been demonstrated to show that the microstructural characteristics may vary the physical characteristics, including surface properties [30].

#### 3.3.3 Surface Roughness

The surface roughness of SFRC resin has been evaluated in seven in vitro experiments [19, 25, 32, 35, 36, 37, 38] and two clinical trials [9, 39]. Reported determinants of surface roughness include filler particles [25, 35, 36] resin matrix composition [32, 35, 36], air bubbles [35, 36], fibre exposure at the surface [35], and fibre hydrolysis [19, 32]. Clinical studies suggest that surface roughness may contribute to restorative failure, although it does not appear to be the primary cause [9, 39].

Resin matrix composition and filler properties influence the surface roughness of SFRC resin [19, 35, 36], similar to reports for PFC resins [36]. Absorption of liquids may increase stress on the fibre‐silane‐matrix interface, which may promote detachment of inorganic particles from the resin surface and result in increased surface roughness [19]. Fibre hydrolysis may contribute to increased surface roughness in SFRC resin, although filler properties appear to have a greater overall influence [19, 32]. Surface modifications such as polishing [35, 37], sandblasting [38], or the presence of air bubbles [36] in cured SFRC resin may expose fibres and increase surface roughness [36]. In clinical applications, manufacturers recommend capping posterior and flowable SFRC resin restorations with a conventional PFC resin layer [8, 9].

Surface roughness increases as wear increases [25]. Surface roughness has also been linked to gloss, as roughness decreases, gloss increases [25].

#### 3.3.4 Water Sorption and Solubility

According to manufacturer data, SFRC resins may exhibit lower water sorption than some PFC resins [40]. In vitro studies found that filler characteristics, surface porosity, and the presence of short fibres may influence water sorption and solubility [35, 41]. Filler characteristics and surface porosity appear to affect these properties in SFRC resin in a similar manner to that reported for PFC resins [35, 41]. Capillary action of fibres, possibly facilitated by interfacial gaps between fibres and the polymer matrix, has been proposed as one mechanism that may increase water sorption in SFRC resins [41].

In one study, the inclusion of short glass fibres within the same resin system had a negligible effect on water sorption and solubility, suggesting that these properties were influenced more by the resin matrix and filler characteristics than by fibre addition [21].

#### 3.3.5 Surface Energy

Surface energy is known to affect the bacterial adhesion of composite resins [35]. One study directly investigated surface free energy of SFRC resins [38], and two studies examined bacterial adhesion on SFRC resin surfaces [35, 36]. In the direct study, surface roughness and contact angle were measured, and surface free energy was calculated using several models [38]. Surface free energy differed among the tested SFRC resins and the comparator flowable PFC resins [38]. Sandblasting was reported to increase the surface free energy of both PFC resins and SFRC resins and produced a more hydrophobic outer surface [38]. Bacterial adhesion to SFRC resin has also been investigated, with one study reporting similar *S. mutans* adhesion for SFRC resin and comparator PFC resin materials [36], and a second study reporting no difference in adhesion between exposed fibres and the adjacent matrix in SFRC resin [35].

## 4 Discussion

### 4.1 Review of the Articles Collected

This review synthesised evidence from in vitro studies (*n* = 28), clinical studies (*n* = 2), and manufacturers' product information (*n* = 3) to address the following question: ‘Does the inclusion of short fibres in resins affect the physical and mechanical surface properties in composite resins when used for direct dental restorations?’ Addressing this question provided insights into the physical and mechanical surface properties of SFRC resins when used for complete replacement of odontogenic tissues. Surface hardness, wear resistance, surface roughness, water sorption and solubility, and surface free energy were found to be the properties that had been investigated.

The grey literature searches found manufacturers' product information (*n* = 3) and published literature references (*n* = 403). However, the three manufacturers' product information sources did not provide sufficient methodological details, limiting transparency and precluding a meaningful risk‐of‐bias assessment.

The published literature was generally judged to have a medium risk of bias, which limited comparability across studies. This was primarily due to inadequate reporting of sample size calculations, operator details, and outcome assessor details. Furthermore, most articles (*n* = 28) provided insufficient information on blinding and randomisation, further restricting meaningful comparisons.

### 4.2 Surface Hardness

In general, the surface hardness of SFRC resins was reported to be equivalent to or greater than that of PFC resins and maintained hardness at greater depths in a laboratory setting [8, 12, 13, 14, 15, 16, 22, 23, 24, 25, 26]. This has partially been attributed to fibre‐assisted light transmission that allows greater light penetration and subsequent higher degree of monomer‐to‐polymer conversion, causing increased hardness [20, 21]. Many factors, including resin matrix composition, filler loading, fibre length/orientation, curing protocol, ageing/storage conditions, and type of photo‐initiator, also influence the surface hardness of SFRC resin [14, 17, 18, 19, 20, 21, 23, 25, 26]. Resin matrix material and filler particle properties also appear to affect surface hardness and surface roughness of SFRC resins [19].

In one study, reduced surface hardness of a SFRC resin was tentatively attributed to the presence of an oxygen‐inhibited layer (OIL), a superficial layer of unpolymerized resin [18]. Exposure to water promotes fibre‐facilitated capillary action which increases water content within the partially soluble resin matrix, thereby decreasing surface hardness [18, 19, 24, 41]. Surface hardness, along with other physical and mechanical properties, is influenced by filler properties [17, 18, 19, 20, 27]. However, the included studies did not report the position of the hardness indenter relative to the filler particles and fibres. It is known that indentation can be dominated by the particles in the composite resin due to differences in size and shape [42]. In SFRC resins, the measured surface hardness may vary according to the phase in contact with the microindenter, that is, resin matrix, particulate fillers, or exposed fibres. In particular, when the indenter contacts exposed glass fibres, higher hardness values may be recorded because the fibres are a harder/stiffer phase than the surrounding resin matrix [43]. Hardness may also depend on fibre type and architecture, as many properties of SFRC resins are influenced by parameters such as fibre diameter, length, orientation and loading [30]. In addition, indentation in SFRC resins may be affected by the spatial distribution of constituents around the indenter due to mesoscale effects [43]. Furthermore, the viscoelastic effect of the resin matrix may cause a decrease in the recorded hardness of the resin composite [42]. Therefore, it can be seen that filler properties and the resin matrix dictate the hardness of composite resins, and the interactions between these two aspects may cause significant changes to the recorded surface hardness values [42]. The increased hardness of SFRC resins may be due to the indentation on the fibre and filler particles rather than an increased surface hardness of the composite resin [42]. This may explain the variability of the hardness reported [26].

Concerns about the exposure of SFRC resins to the oral environment have led manufacturers to recommend surface coverage with PFC resins of these materials; however, the available in vitro evidence suggests that some SFRC resins may achieve surface hardness compatible with use as exposed direct dental restorative materials [16]. However, one study found no relationship between hardness and wear [25], suggesting that other surface properties are arguably more important in the exposure of SFRC resins.

### 4.3 Wear

Surface wear of SFRC resins has been investigated extensively in vitro using a range of approaches [18, 25, 28, 29, 30, 31, 32] and in one clinical study [9]. The results consistently suggest that surface fibre plucking is a common phenomenon in SFRC resins [34]. Clinical surface wear of composite resins is related to attrition, erosion, and abrasion [32]. SFRC resins are expected to exhibit similar clinical characteristics [32]. However, the effect on the adjacent and opposing tooth structures and replacement materials of SFRC resin has not been well studied to date.

Several factors influence the wear resistance of SFRC resins, including random fibre orientation [29, 31], and reduced surface plucking of fibres [28, 30, 34]. Whereas excess fibres at the surface increase fibre plucking, causing greater SFRC resins surface wear [1, 34]. Similarly, the wear resistance of SFRC resins is affected by fibre length [32]. Optimal fibre length appears to be important for the wear resistance of SFRC resin. Some evidence suggests that longer fibres are associated with greater surface wear resistance than shorter fibres [34]. Fibres are only beneficial to wear resistance when they exceed the critical fibre length, which may be as much as 50 times the fibre diameter, despite variations of the fibre matrix interface [19, 31, 34].

Surface wear of SFRC resins is affected by environmental conditions; pH cycling can accelerate wear [32], whereas hydrothermal ageing did not affect wear [18]. Increased instability of the monomer amplifies wear of SFRC resin [30].

Moreover, variations in filler characteristics, including fibre dimensions and matrix monomer composition, may affect the filler‐matrix interface and cause changed wear rates and patterns [25, 32]. This has clinical implications for the use of SFRC resins due to the relationship between critical fibre length and surface wear, depending on the energy transfer from the exposed fibre to the resin matrix [28, 30]. One in vitro study justified that Ever X flow (~5‐6 μm diameter and 200‐300 μm length) displayed superior surface characteristics compared to Ever X posterior (~17 μm diameter and 800 ~ μm length) [30]. Both materials display similar critical fibre length and aspect ratio. Lassila et al. reported that Ever X flow exhibited the lowest wear depth among the tested SFRC resins and noted SFRC resin properties are strongly influenced by parameters such as fibre diameter, length, orientation and loading. A clinical study showed that Ever X flow had sufficient surface properties to be used in the interproximal region [9]. Taken together, these findings are consistent with manufacturer recommendations, whereby SFRC resins containing larger fibres, such as Ever X posterior, should generally be capped with a PFC resin.

Another important aspect regarding the clinical acceptance of wear is the relationship between wear and other surface and bulk properties. One study found that wear affects roughness, which affects gloss [25]. Another study investigated marginal wear of SFRC resin; although not within the search criteria for this review this work established although not within the criteria of the search criteria for this review, this work established that marginal wear of SFRC resins in both enamel and ceramic restorations to be sufficient despite differences in wear rate [44]. Furthermore, the study suggested SFRC resins may be suitable as a possible repair material for ceramic restorations [44].

Hence, manufacturers have held concerns about the high surface wear characteristics of SFRC resins. However, manufacturers have mostly been concerned about the exposure of fibres to the oral environment and so have suggested the coverage of PFC resins [8]. Although this may be true for SFRC resins with larger fibre dimensions, there is increasing evidence that the wear of exposed SFRC resins is satisfactory for clinical use.

### 4.4 Surface Roughness

Surface roughness of SFRC resins is affected by both filler particles [25, 35, 36] and resin matrices [32, 35, 36]. Moreover, surface treatments and structural defects—such as polishing [25, 35, 37], sandblasting [38], and the presence of air bubbles [36], may result in fibre exposure and increased surface roughness. Exposed fibres have been indirectly linked to increased roughness [9, 32]. The surface roughness of SFRC resin has been examined through in vitro experiments [19, 35, 36, 37, 38], and clinical studies [9, 39]. Clinical investigations (*n* = 2) have assessed the surfaces of SFRC resin restorations and suggested that surface roughness was not a primary cause of failure but may be a contributing factor [9, 39]. In vitro studies demonstrated that SFRC resins have higher surface roughness compared to their PFC resin counterparts; however, the results remain within the clinically accepted surface roughness (Ra) value of 0.26–0.42 μm [34].

Two main mechanisms explain how surface roughness may affect the longevity of composite resin restorations: (i) direct loss of tooth structure and restorative materials [32, 33], and (ii) increased bacterial adhesion, which may contribute to future carious lesions at restoration margins [35, 36, 37].

The direct loss of tooth structure may result from repeated contact of the SFRC resin to opposing or adjacent structures [33, 34]. An increased surface roughness can also lead to increased abrasion that can cause early failure of the placed restoration [32]. Hydrolysis contributes to increased surface roughness by water sorption softening the resin matrix and weakening the filler‐matrix interface, promoting loss of filler particles [19]. Water‐mediated hydrolysis of SFRC resin may alter the abrasive response of SFRC resin by reducing hardness [19], although the mechanism behind this remains unclear.

The free surface energy of a composite resin is linked to an increase in bacterial adhesion [35, 36]. The presence of glass fibres increases the surface free energy of composite resins and so it would be expected that bacterial adhesion would increase. Some studies have reported greater bacterial adhesion on certain SFRC resins compared with PFC resins [35], although this has not been a consistent finding across all materials studied [35, 36]. One study found that the bacteria are not selective for the increased surface energy of the fibres and are non‐selective between the polymer matrix and the filler particles [35]. Based on this, bacterial adhesion on SFRC resin does not appear to be determined solely by the presence or absence of fibres [35]. Instead, surface alterations unique to SFRC resins affect bacterial adhesion, such as fibre plucking [34] and hydrolysis [19], have a greater effect on the bacterial adhesion to the surface of SFRC resins by increasing the surface roughness. These phenomena may increase the surface roughness but are not the only factors involved in surface bacterial adhesion.

SFRC resin shows clinically acceptable surface roughness when the surface is left unaltered after curing [35, 37, 38], despite concerns of exposed fibres. If surface alteration or surface defects are present, then the roughness may exceed safe clinical limits [35]. Increased roughness enhances bacterial adhesion and possibly the effect of wear, which may contribute to the clinical failure of SFRC resin restorations [9, 32]. However, this has not been reported for SFRC resins with smaller fibre dimensions [35].

Other studies have investigated the surface gloss of SFRC resin and other restorative resin composites [25, 45]. Available evidence indicates surface gloss is generally inversely related to surface roughness, although the strength of this relationship varies with material composition and polishing protocol [45]. In short‐fibre‐reinforced CAD/CAM composite blocks, incorporation of fibres did not compromise surface quality, as polished samples showed low roughness and high gloss, with no evidence of microfibre protrusion [25].

### 4.5 Water Sorption and Solubility

Once polymerised, the resin matrix in a SFRC resin is more stable and less hydrophilic than its unpolymerized monomer components [16]. Water sorption is the uptake of water molecules into the composite resin matrix and polymer network, a process that can lead to mechanical failure of the composite resin [40, 41]. Filler properties influence water sorption through the hydrophilicity and chemical stability of the resin matrix [29], and affect the degree of conversion and, therefore, the water sorption [29]. However, filler particles exert greater influence on the degree of conversion and hydrophilicity than the presence of short fibres [29]. Thus, filler particles play a more significant role in determining the water sorption of SFRC resins than the short fibres.

Water solubility refers to the loss of monomers and other chemical compounds from a composite resin into the surrounding aqueous environment [41]. The fibres have little or no effect on water sorption, especially at greater depths [18, 21, 41]. Whereas filler properties have a more pronounced effect on the water sorption of SFRC resins than the presence of fibres [29, 41]. Current evidence suggests that ethanol may increase the solubility of SFRC resins, potentially through the capillary action of the short fibres and subsequent hydrolytic degradation, although the clinical significance of this is uncertain [19, 41].

### 4.6 Surface Energy

A small number of studies (*n* = 3) have investigated the effects of short fibres on the surface energy of composite resin [35, 36, 38]. SFRC resins have been reported to have higher free surface energy units compared to PFC resins [38]. Sandblasting is known to increase the surface free energy of PFC resin and SFRC resins, although its clinical significance has yet to be established [38]. In vitro studies investigating bacterial adhesion suggest that the surface free energy of SFRC resins does not affect bacterial adhesion, unlike PFC resins [35, 36]. This suggests that, despite the higher surface free energy of SFRC resin, limited clinical impact is expected.

## 5 Conclusions

This review synthesised the available literature on the physical and mechanical surface properties of SFRC resins, including surface hardness, surface roughness, wear, water sorption/solubility, and surface energy. Taken together, the available evidence suggests that some SFRC resins may be compatible with uncapped use in the oral environment. However, this is likely to be material‐dependent, as the surface properties of SFRC resin are affected by filler characteristics, polymer matrix composition, fibre characteristics, fibre‐silane‐matrix bonding, and surface treatment. In general, smaller fibre dimensions that exceed the critical fibre length show more favourable characteristics to allow the exposure of fibres to the oral environment. Based on the reviewed data, incorporating short fibres into composite resins to produce SFRC resins does not appear to affect surface properties to an extent that would preclude the clinical use of exposed SFRC resin in direct restorations. Further clinical studies are required to confirm or challenge these findings.

## Author Contributions

**Michael Liddell:** writing – review and editing, supervision, conceptualization. **Murray Macpherson:** investigation, writing – original draft, writing – review and editing, methodology, project administration, formal analysis. **Touraj Nejatian:** conceptualization, writing – review and editing, supervision.

## Funding

The authors have nothing to report.

## Ethics Statement

The authors have nothing to report.

## Conflicts of Interest

The authors declare no conflicts of interest.

## Supporting information


**Data S1:** adj70051‐sup‐0001‐DataS1.docx.
**Appendix 1**. QUIN (Quality Assessment for In‐Vitro Studies) tool criteria.
**Appendix 2**. QUIN assessment tool results.
**Appendix 3**. Extracted data from reviewed articles.

## Data Availability

The data that support the findings of this systematic review are derived from published studies, all of which are cited in the reference list. No supplementary data files are associated with this manuscript, and no additional data were used to support the study.

## References

[1] M. Kimmel and C. Faggion , “Systematic Reviews Comparing Direct and Indirect Restorations: An Umbrella Review That Examines Restoration Type and Confidence in Results,” Clinical and Experimental Dental Research 11, no. 3 (2025): e70149, 10.1002/cre2.70149. 40417986 PMC12104983

[2] R. Sakaguchi , J. Ferracane , and J. Powers , “Chapter 1, Role and Significance of Restorative Dental Materials,” in Craig's Restorative Dental Materials, 14th ed. (Elsevier Inc., 2019), 1–3.

[3] J. Matos , L. Nakano , G. Lopes , et al., “Characterization of Bulk‐Fill Resin Composites in Terms of Physical, Chemical, Mechanical and Optical Properties and Clinical Behavior,” Journal of Forensic Odonto‐Stomatology 15 (2021): 226–233, https://ijodontostomatology.com/en/articulo/characterization‐of‐bulk‐fill‐resin‐composites‐in‐terms‐of‐physical‐chemical‐mechanical‐and‐optical‐properties‐and‐clinical‐behavior/.

[4] S. Schricker , “Composite Resin Polymerization and Relevant Parameters,” in Orthodontic Applications of Biomaterials, 1st ed., ed. T. Eliades and W. Brantley (Woodhead Publishing, 2017), 153–170, 10.1016/B978-0-08-100383-1.00009-6.

[5] S. Garoushi , A. Gargoum , P. Vallittu , and L. Lassila , “Short Fiber‐Reinforced Composite Restorations: A Review of the Current Literature,” Journal of Investigative and Clinical Dentistry 9 (2018): e12330, 10.1111/jicd.12330. 29479830

[6] K. Cho , G. Wang , R. Raju , G. Rajan , J. Fang , and M. Stenzel , “Influence of Surface Treatment on the Interfacial and Mechanical Properties of Short s‐Glass Fiber‐Reinforced Dental Composites,” ACS Applied Materials & Interfaces 11, no. 35 (2019): 32328–32338, 10.1021/acsami.9b01857. 31393104

[7] “Ever X Flow From GC—Short‐Fibre Reinforced Composite for Dentine Replacement. GC Materials. GC Materials,” (2023), https://www.gc.dental/australasia/sites/australasia.gc.dental/files/products/downloads/everxflow/literature‐list/literature‐list‐evderx‐flow.pdf.

[8] “EverX Posterior & EverX Flow Study Compilation. GC Materials,” (2024), https://www.gc.dental/europe/sites/europe.gc.dental/files/products/downloads/everxflow/reference/REF_World_of_Proof_‐_Study_Compilation.pdf.

[9] R. ElAziz , S. ElAziz , P. ElAziz , et al., “Clinical Evaluation of Posterior Flowable Short Fiber‐Reinforced Composite Restorations Without Proximal Surface Coverage,” Odontology 112, no. 4 (2024): 1274–1283, 10.1007/s10266-024-00905-5. 38393515 PMC11415407

[10] A. Liberati , D. Altman , J. Tetzlaff , et al., “The PRISMA Statement for Reporting Systematic Reviews and Meta‐Analyses of Studies That Evaluate Health Care Interventions: Explanation and Elaboration,” PLoS Medicine 6 (2009): e1000100, 10.1371/journal.pmed.1000100. 19621070 PMC2707010

[11] V. Sheth , N. Shah , R. Jain , N. Bhanushali , and V. Bhatnagar , “Development and Validation of a Risk‐Of‐Bias Tool for Assessing In Vitro Studies Conducted in Dentistry: The QUIN,” Journal of Prosthetic Dentistry 131, no. 6 (2024): 1038–1042. 35752496 10.1016/j.prosdent.2022.05.019

[12] C. Hambire , U. Hambire , and S. Shirsath , “Comparison and Optimization of Wear Rates of Two Types of Dental Composites on the Basis of Micro Hardness,” International Journal of Engineering Science Invention 3, no. 10 (2014): 1–5, https://www.ijesi.org/papers/Vol(3)10/A031010105.pdf.

[13] V. Miletic , P. Pongprueksa , J. De Munck , N. Brooks , and B. Van Meerbeek , “Curing Characteristics of Flowable and Sculptable Bulk‐Fill Composites,” Clinical Oral Investigations 21, no. 4 (2017): 1201–1212, 10.1007/s00784-016-1894-0. 27383375

[14] S. Garoushi , P. Vallittu , and L. Lassila , “Effect of Particulate Nanofiller on the Surface Microhardness of Glass Fiber Reinforced Filling Composite,” Chinese Journal of Dental Research 11, no. 1 (2008): 20–24, https://www.researchgate.net/publication/302910180_Effect_of_Particulate_Nanofillers_on_the_Surface_Microhardness_of_Glass‐Fibre‐Reinforced_Filling_Composite_Resin.

[15] A. Samuel , R. Raju , K. Sreejith , B. Kalathil , D. Nenavath , and V. Chaitra , “Comparative Evaluation of the Surface Hardness of Different Esthetic Restorative Materials: An In Vitro Study,” Journal of Pharmacy & Bioallied Sciences 12, no. Suppl 1 (2020): S124–S128, 10.4103/jpbs.JPBS_40_20. 33149442 PMC7595552

[16] A. Alshabib , C. Jurado , F. Azpiazu‐Flores , K. Aldosary , A. Tsujimoto , and H. Algamaiah , “Mechanical Properties and Degree of Conversion of Resin‐Based Core Build‐Up Materials and Short Fiber‐Reinforced Flowable Resin‐Based Composite,” Dental Materials Journal 43, no. 3 (2024): 453–459, 10.4012/dmj.2023-207. 38692907

[17] A. Alshabib , N. Silikas , and D. Watts , “Hardness and Fracture Toughness of Resin‐Composite Materials With and Without Fibers,” Dental Materials 35, no. 8 (2019): 1194–1203, 10.1016/j.dental.2019.05.017. 31176452

[18] J. Oja , L. Lassila , P. Vallittu , and S. Garoushi , “Effect of Accelerated Aging on Some Mechanical Properties and Wear of Different Commercial Dental Resin Composites,” Materials (Basel) 14, no. 11 (2021): 2769, 10.3390/ma14112769. 34071137 PMC8197073

[19] C. Kumari , K. Bhat , R. Bansal , N. Singh , A. Anupama , and T. Lavanya , “Evaluation of Surface Roughness and Hardness of Newer Nanoposterior Composite Resins After Immersion in Food‐Simulating Liquids,” Contemporary Clinical Dentistry 10, no. 2 (2019): 289–293, 10.4103/ccd.ccd_535_18. 32308292 PMC7145263

[20] S. Garoushi , P. Vallittu , A. Shinya , and L. Lassila , “Influence of Increment Thickness on Light Transmission, Degree of Conversion and Micro Hardness of Bulk Fill Composites,” Odontology 104, no. 3 (2016): 291–297, 10.1007/s10266-015-0227-0. 26660101

[21] R. Raju , G. Rajan , P. Farrar , and G. Prusty , “Dimensional Stability of Short Fibre Reinforced Flowable Dental Composites,” Scientific Reports 11, no. 1 (2021): 4697, 10.1038/s41598-021-83947-x. 33633198 PMC7907147

[22] H. Abouelleil , N. Pradelle , C. Villat , N. Attik , P. Colon , and B. Grosgogeat , “Comparison of Mechanical Properties of a New Fiber Reinforced Composite and Bulk Filling Composites,” Restorative Dentistry and Endodontics 40, no. 4 (2015): 262–270, 10.5395/rde.2015.40.4.262. 26587411 PMC4650521

[23] N. Attik , P. Colon , R. Gauthier , C. Chevalier , B. Grosgogeat , and H. Abouelleil , “Comparison of Physical and Biological Properties of a Flowable Fiber Reinforced and Bulk Filling Composites,” Dental Materials 38, no. 2 (2022): e19–e30, 10.1016/j.dental.2021.12.029. 34961643

[24] S. Garoushi , P. Vallittu , and L. Lassila , “Depth of Cure and Surface Microhardness of Experimental Short Fiber‐Reinforced Composite,” Acta Odontologica Scandinavica 66, no. 1 (2008): 38–42, 10.1080/00016350801918377. 18320417

[25] E. Mangoush , L. Lassila , P. K. Vallittu , and S. Garoushi , “Microstructure and Surface Characteristics of Short‐Fiber Reinforced CAD/CAM Composite Blocks,” European Journal of Prosthodontics and Restorative Dentistry 29, no. 3 (2021): 166–174, 10.1922/EJPRD_2207Mangoush09. 33508179

[26] C. Meenakumari , K. Bhat , R. Bansal , and N. Singh , “Evaluation of Mechanical Properties of Newer Nanoposterior Restorative Resin Composites: An In Vitro Study,” Contemporary Clinical Dentistry 9, no. Suppl 1 (2018): S142–S146, 10.4103/ccd.ccd_160_18. 29962780 PMC6006874

[27] M. Fráter , J. Grosz , A. Jakab , et al., “Evaluation of Microhardness of Short Fiber‐Reinforced Composites Inside the Root Canal After Different Light Curing Methods – An In Vitro Study,” Journal of the Mechanical Behavior of Biomedical Materials 150 (2024): 106324, 10.1016/j.jmbbm.2023.106324. 38113823

[28] S. Garoushi , E. Säilynoja , M. Frater , F. Keulemans , P. K. Vallittu , and L. Lassila , “A Comparative Evaluation of Commercially Available Short Fiber‐Reinforced Composites,” BMC Oral Health 24, no. 1 (2024): 1573, 10.1186/s12903-024-05267-6. 39736654 PMC11684104

[29] L. Lassila , E. Säilynoja , R. Prinssi , P. Vallittu , and S. Garoushi , “Characterization of a New Fiber‐Reinforced Flowable Composite,” Odontology 107, no. 3 (2019): 342–352, 10.1007/s10266-018-0405-y. 30617664 PMC6557871

[30] L. Lassila , F. Keulemans , P. Vallittu , and S. Garoushi , “Characterization of Restorative Short‐Fiber Reinforced Dental Composites,” Dental Materials Journal 39, no. 6 (2020): 992–999, 10.4012/dmj.2019-088. 32779605

[31] S. Garoushi , P. Vallittu , and L. Lassila , “Mechanical Properties and Wear of Five Commercial Fibre‐Reinforced Filling Materials,” Chinese Journal of Dental Research 20, no. 3 (2017): 137–143, 10.3290/j.cjdr.a38768. 28808697

[32] F. Asadian , Z. Shahidi , and Z. Moradi , “Evaluation of Wear Properties of Four Bulk‐Fill Composites: Attrition, Erosion, and Abrasion,” BioMed Research International 2021 (2021): 8649616, 10.1155/2021/8649616. 34805405 PMC8604596

[33] A. Kumar , A. Sarthaj , and D. Majumder , “Comparative Evaluation of Wear Resistance of Cast Gold With Bulk‐Fill Composites an In Vitro Study,” Journal of Conservative Dentistry 21, no. 3 (2018): 302–305, 10.4103/JCD.JCD_196_17. 29899634 PMC5977780

[34] D. Callaghan , A. Vaziri , and H. Nayeb‐Hashemi , “Effect of Fiber Volume Fraction and Length on the Wear Characteristics of Glass Fiber‐Reinforced Dental Composites,” Dental Materials 22, no. 1 (2006): 84–93, 10.1016/j.dental.2005.02.011. 16002133

[35] L. Lassila , V. Loimaranta , P. Vallittu , and S. Garoushi , “Bacterial Adhesion and Surface Roughness of Particulate‐Filled and Short Fiber‐Reinforced Composites,” Odontology 113, no. 2 (2025): 634–644, 10.1007/s10266-024-00997-z. 39316233 PMC11950134

[36] L. Lassila , S. Garoushi , J. Tanner , P. Vallittu , and E. Söderling , “Adherence of *Streptococcus mutans* to Fiber‐Reinforced Filling Composite and Conventional Restorative Materials,” Open Dentistry Journal 3 (2009): 227–232, 10.2174/1874210600903010227. 20148170 PMC2817876

[37] S. Jafarnia , A. Valanezhad , S. Shahabi , S. Abe , and I. Watanabe , “Physical and Mechanical Characteristics of Short Fiber‐Reinforced Resin Composite in Comparison With Bulk‐Fill Composites,” Journal of Oral Science 63, no. 2 (2021): 148–151, 10.2334/josnusd.20-0436. 33504755

[38] S. Łagan , A. Chojnacka‐Brożek , A. Liber‐Kneć , and G. Malik , “Surface Free Energy and Roughness of Flowable Dental Composites,” Acta of Bioengineering and Biomechanics 25, no. 3 (2023): 61–71, 10.37190/ABB-02306-2023-02.38314525

[39] J. van Dijken and K. Sunnegårdh‐Grönberg , “Fiber‐Reinforced Packable Resin Composites in Class II Cavities,” Journal of Dentistry 34, no. 10 (2006): 763–769, 10.1016/j.jdent.2006.02.003. 16580114

[40] NovaPro , “The First and Only Nanofiber Reinforced Universal and Flowable Composite,” (2025), https://www.pearsondental.com/flyers/Sell‐Sheet‐NovaPro.pdf.

[41] A. Alshabib , N. Silikas , H. Algamaiah , et al., “Effect of Fibres on Physico‐Mechanical Properties of Bulk‐Fill Resin Composites,” Polymers (Basel) 15, no. 16 (2023): 3452, 10.3390/polym15163452. 37631507 PMC10457899

[42] R. B. Price and B. Sullivan , “Effect of Indenter Load on Vickers Microhardness and Indentation Depth of One Resin Composite,” Materials (Basel) 17, no. 24 (2024): 6156, 10.3390/ma17246156. 39769756 PMC11678071

[43] N. Rauter and R. Lammering , “Experimental Characterization of Short Fiber‐Reinforced Composites on the Mesoscale by Indentation Tests,” Applied Composite Materials 28, no. 4 (2021): 1747–1765, 10.1007/s10443-021-09937-4.

[44] L. Lassila , R. Novotny , E. Säilynoja , P. K. Vallittu , and S. Garoushi , “Wear Behavior at Margins of Direct Composite With CAD/CAM Composite and Enamel,” Clinical Oral Investigations 27, no. 5 (2023): 2419–2426, 10.1007/s00784-023-04883-w. 36746817 PMC10159970

[45] L. Lassila , E. Säilynoja , R. Prinssi , P. K. Vallittu , and S. Garoushi , “The Effect of Polishing Protocol on Surface Gloss of Different Restorative Resin Composites,” Biomaterial Investigations in Dentistry 7, no. 1 (2020): 1–8, 10.1080/26415275.2019.1708201. 32010900 PMC6968704

