# Supplementary material for: Surface Physical and Mechanical Properties of Short Fibre Reinforced Composite Resins in Direct Restorative Dentistry: A Systematic Review
Source: Aust Dent J. 2026 May 11;71(3):221–30. doi: 10.1111/adj.70051 (PMC13506663; doi:10.1111/adj.70051)
Supplement: Supplementary file 1 [file ADJ-71-221-s001.docx]

8.0 Appendix

**Appendix 1**: QUIN (Quality Assessment for In-Vitro Studies) tool criteria.

| **Criteria** | | **Details** |
| --- | --- | --- |
| 1 | Clearly stated aims/objectives | Study should clearly state aims and/or objectives, which should then be followed throughout. |
| 2 | Detailed explanation of sample size calculation | Details regarding method by which given sample size calculated should be clearly stated. Details regarding software program, formula, and parameters used for calculation of sample size should also be specified. |
| 3 | Detailed explanation of sampling technique | Details regarding predefined population from which sample has been selected. Details of sampling technique and inclusion and exclusion criteria should be clearly stated. |
| 4 | Details of comparison group | Details of comparison group (positive control, negative control, or standard) should be clearly specified. |
| 5 | Detailed explanation of methodology | Clarity of procedure, method of standardization, and details of any universal standards used (if applicable) should be clearly stated. |
| 6 | Operator details | Number of operators and details regarding training and calibration of operator/s (inter-operator and intra-operator reliability) should be clearly specified. |
| 7 | Randomization | Details regarding sequence generation and allocation concealment should be clearly stated. |
| 8 | Method of measurement of outcome | Clarity of procedure and rationale for choosing method should be stated. Method of standardization along with details of any universal standards used (if applicable) should also be clearly specified. |
| 9 | Outcome assessor details | Number of outcome assessors and details regarding training and calibration of assessor/s (inter-outcome and intra-outcome assessor reliability) should be clearly specified. |
| 10 | Blinding | Details regarding blinding of operator(s), outcome assessor(s), and statistician should be clearly specified. |
| 11 | Statistical analysis | Details regarding software program used and statistical analysis should be clearly specified. |
| 12 | Presentation of results | Outcome should be based on predefined aims and/or objectives. All data should be adequately tabulated with baseline data clearly specified (if applicable). |

**Appendix 2:** QUIN assessment tool results

| **Study** | **QUIN tool assessment criterion** | | | | | | | | | | | | **Total score** | **Final score (%)** | **Risk level** |
| --- | --- | --- | --- | --- | --- | --- | --- | --- | --- | --- | --- | --- | --- | --- | --- |
|  | **1** | **2** | **3** | **4** | **5** | **6** | **7** | **8** | **9** | **10** | **11** | **12** |  |  |  |
| GC Materials. (2023)^7^ | - | - | - | - | - | - | - | - | - | - | - | - | 0 | - | - |
| GC Materials. (2024)^8^ | - | - | - | - | - | - | - | - | - | - | - | - | 0 | - | - |
| ElAziz et al. (2024)^9^ | 1 | 2 | 2 | 2 | 2 | 2 | 2 | 1 | 2 | 2 | 2 | 2 | 22 | 91.7 | Low |
| Hambire et al. (2014)^12^ | 1 | 0 | 2 | 2 | 1 | 0 | - | 2 | 0 | - | 0 | 2 | 10 | 50 | Medium |
| Miletic et al. (2016)^13^ | 2 | 0 | 2 | 2 | 2 | 0 | - | 2 | 0 | - | 2 | 2 | 14 | 70 | Medium |
| Garoushi et al. (2008)^14^ | 2 | 0 | 2 | 2 | 2 | 0 | - | 2 | 0 | - | 2 | 2 | 14 | 70 | Medium |
| Samuel et al. (2020)^15^ | 1 | 1 | 2 | 2 | 2 | 0 | - | 1 | 0 | - | 2 | 1 | 12 | 60 | Medium |
| Alshabib et al. (2024)^16^ | 2 | 2 | 2 | 2 | 2 | 0 | - | 2 | 0 | - | 2 | 2 | 16 | 80 | Medium |
| Alshabib et al. (2019)^17^ | 2 | 0 | 2 | 1 | 2 | 2 | 0 | 2 | 2 | 0 | 2 | 2 | 17 | 70.8 | Low |
| Oja et al. (2021)^18^ | 2 | 0 | 2 | 2 | 2 | 0 | - | 2 | 0 | - | 2 | 2 | 14 | 70 | Medium |
| Kumari et al. (2019)^19^ | 2 | 2 | 2 | 2 | 2 | 0 | - | 2 | 1 | - | 2 | 2 | 17 | 85 | Low |
| Garoushi et al. (2015)^20^ | 2 | 0 | 0 | 2 | 2 | 0 | - | 2 | 0 | - | 2 | 2 | 12 | 60 | Medium |
| Raju et al. (2021)^21^ | 2 | 0 | 2 | 1 | 2 | 0 | - | 2 | 0 | - | 2 | 2 | 13 | 65 | Medium |
| Abouelleil et al. (2015)^22^ | 1 | 0 | 2 | 2 | 2 | 0 | - | 1 | 0 | - | 2 | 2 | 12 | 60 | Medium |
| Attik et al. (2022)^23^ | 1 | 0 | 2 | 2 | 1 | 0 | - | 1 | 0 | - | 2 | 2 | 11 | 55 | Medium |
| Garoushi et al. (2008)^24^ | 2 | 0 | 2 | 2 | 2 | 0 | - | 2 | 0 | - | 2 | 2 | 14 | 70 | Medium |
| Mangoush et al. (2021)^25^ | 2 | 0 | 1 | 2 | 1 | 0 | - | 2 | 0 | - | 2 | 2 | 12 | 60 | Medium |
| Meenakumari et al. (2018)^26^ | 2 | 0 | 2 | 2 | 2 | 0 | - | 2 | 0 | - | 2 | 2 | 14 | 70 | Medium |
| Fráter et al. (2023)^27^ | 2 | 0 | 2 | 2 | 2 | 0 | - | 2 | 0 | - | 1 | 2 | 13 | 65 | Medium |
| Garoushi et al. (2024)^28^ | 2 | 1 | 2 | 2 | 1 | 0 | - | 1 | 0 | - | 2 | 2 | 13 | 65 | Medium |
| Lassila et al. (2019)^29^ | 2 | 0 | 2 | 2 | 2 | 0 | - | 1 | 0 | - | 2 | 2 | 13 | 65 | Medium |
| Lassila et al. (2020)^30^ | 2 | 0 | 2 | 2 | 2 | 0 | - | 2 | 0 | - | 2 | 2 | 14 | 70 | Medium |
| Garoushi et al. (2017)^31^ | 2 | 0 | 1 | 1 | 2 | 0 | - | 2 | 0 | - | 2 | 2 | 12 | 60 | Medium |
| Asadian et al. (2021)^32^ | 2 | 0 | 2 | 0 | 2 | 0 | - | 2 | 0 | - | 2 | 2 | 12 | 60 | Medium |
| Sarthaj et al. (2018)^33^ | 2 | 0 | 2 | 2 | 2 | 0 | - | 2 | 0 | - | 2 | 2 | 14 | 70 | Medium |
| Callaghan et al (2006)^34^ | 2 | 0 | 1 | 1 | 2 | 0 | - | 2 | 0 | - | 0 | 2 | 10 | 50 | Medium |
| Lassila et al. (2024)^35^ | 2 | 1 | 2 | 2 | 2 | 1 | - | 1 | 0 | - | 2 | 2 | 15 | 75 | Low |
| Lassila et al. (2009)^36^ | 2 | 0 | 2 | 2 | 2 | 0 | - | 1 | 0 | - | 2 | 2 | 13 | 65 | Medium |
| Jafarnia et al. (2021)^37^ | 2 | 0 | 2 | 2 | 2 | 0 | - | 2 | 0 | - | 2 | 2 | 14 | 70 | Medium |
| Łagan et al. (2023)^38^ | 2 | 0 | 2 | 2 | 2 | 0 | - | 2 | 0 | - | 2 | 2 | 14 | 70 | Medium |
| van Dijken et al. (2006)^39^ | 2 | 2 | 2 | 2 | 2 | 0 | - | 2 | 0 | - | 2 | 2 | 16 | 80 | Low |
| NovaPro. (n.d.)^40^ | - | - | - | - | - | - | - | - | - | - | - | - | 0 | - | - |
| Alshabib et al. (2023)^41^ | 2 | 0 | 1 | 1 | 2 | 0 | - | 2 | 0 | - | 2 | 2 | 12 | 60 | Medium |

**Appendix 3:** Extracted data from reviewed articles

| **Reference** | **Title** | **Materials used** | **Surface properties investigated** | **Methodology** | **Results** | **Conclusions** |
| --- | --- | --- | --- | --- | --- | --- |
| GC Materials. (2023)^7^ | Reference List | Reference list of multiple studies | Studies investigate multiple characteristics | Un stated | Not given | Not given |
| GC Materials. (2024)^8^ | A world of Proof, Discover the power of fibres | Ever X posterior and Ever X flow | Microhardness | Multiple references | Light cured ever X shows clinically sound hardness up to 4mm of 80% | Better mechanical properties of Ever X than conventional and bulk PFC. Only laboratory studies mentioned. |
|  |  |  | Manufacturer's instructions |  | Surface coverage with conventional Composite resin is the instructed use of ever X posterior and ever x flow |  |
| ElAziz et al. (2024)^9^ | Clinical evaluation of posterior flowable short fiber-reinforced composite restorations without proximal surface coverage | Clinical evaluation of G –aenial Posterior in incremental build-up of class II restorations and Ever X bulk and dentin without proximal coverage | Clinical evaluation of Anatomical contour (wear) | 70 patients treated, 35 with traditional PFC incremental technique, 35 with SFRC without proximal coverage. Immediate, 6-month, and 18-month review. | No clinical difference between the PFC and SFRC | Surface properties of SFRC are clinically sound over 18 months. |
|  |  |  | Clinical evaluation of Surface texture |  | No clinical difference between the PFC and SFRC |  |
| Hambire et al. (2014)^12^ | Comparison and optimization of wear rates of two types of dental composites on the basis of microhardness | ESPE 3M (micro filled) and Ever X posterior (SFRC) | Vickers surface microhardness test | Vicker's Hardness machine used or 10 sec at 25g, 50g, and 100g of force | Ever X is harder than ESPE 3M | Increased hardness leads to less wear. Optimal wear rate to coincide with functional load, anatomical geometry of adjacent teeth, and oral environment experienced by CR. |
|  |  |  | Wear test – Ball on disc | Ball on disc machine used for two body wear test at various cycles, load and ball diameter. | Ever X has less wear than ESPE 3M but, the ball diameter, number of cycles and force applied effect the correlation. |  |
| Miletic et al. (2016)^13^ | Curing characteristics of flowable and sculptable bulk-fill composites | 2 x 2 mm increments cured for 10 sec each, 1 x 4mm increment cured for 10 sec, 1x4mm increment cured for 20 sec, 1 6mm increment cured for 20 sec with Filtek Bulk, SDR, Sonic Fil, Xenius base (SFRC fibre length of about 1.3mm), Tertic Evoceram bulk, and Teric EvoCeram (control) | Vicker’s surface hardness test | Vicker's hardness test 1 kg for 10 sec at x10 magnification | Xenius base is comparable to CR. Top surface hardness of all CR was more consistent than the lower surface. >80% Top bottom hardness ratio was experienced by all composites except conventional. For Xenius base positive correlation between depth of cure and top hardness. All CR correlated bottom surface hardness. | Fibre reinforced composites behave in similar ways to conventional composites the main difference is that the degree of conversion and Hardness are more stable correlations. |
| Garoushi et al. (2008)^14^ | Effect of Particulate Nanofillers on the Surface Microhardness of Glass-Fibre-Reinforced Filling Composite Resin | Enamel and dentin (controls), Composite resins (Z250, Grandio and nulite) FRC (Everstick and Ribbond), RMGIC (FUJI II LC), Amalgam (ANA 2000), Prefabricated ceramic (Cerena Class I) Experimental CR (22.5% Fibres (3mm) to 0,10,20,30,40,50% nanofillers) | Vickers surface microhardness test | Samples were cured and polished then tested with universal Vicker's hardness (1.96N, for 10sec) | Increased quantity of nanofiller increases surface hardness. At 60% the hardness is that of commercial PFC (Z250) and SFRC (nulite). PRC with high filler were harder than SFRCs. FRC had same or lower hardness than SFRC. | High fraction nanofillers with fibres could increase hardness. Other clinical features need to be assessed such as wear. SFRC could be used for posterior restorations, but clinical trials are needed. |
| Samuel et al. (2020)^15^ | Comparative Evaluation of the Surface Hardness of Different Esthetic Restorative Materials: An In Vitro Study | Comparison of surface hardens of filtek bulk fill, Teric Evo Ceram Bulk fill, Ever X posterior and compared to each other. | Vicker’s surface hardness test | Vicker’s hardness test completed with 50g for 15 sec on the top and bottom of samples (2mm thick and 8mm in diameter) | Ever X posterior had the highest hardness of all composites at 0 and 2mm followed by BEUTIFUL bulk and Teric Ceram Bulk | Ever X posterior has higher hardness. Clinical studies are needed. |
| Alshabib et al. (2024)^16^ | Mechanical properties and degree of conversion of resin-based core build-up materials and short fiber-reinforced flowable resin-based composite | Dual cure: multi core flow, activa, core X. SFCR: ever X posterior | Vickers surface Microhardness test | Mylar strip on surface, and cure. 1h, 30-day water immersion then Vicker’s hardness | All materials except ever X had increased hardness after 30 days in distilled water. | Hardness is linked to degree of conversion. Surface hardness is not the only property that needs to be examined for clinical acceptance. |
| Alshabib et al. (2019)^17^ | Hardness and fracture toughness of resin-composite materials with and without fibers | SFRC-(NovaPro universal, NovaPro Flow, Ever X posterior) PFC -(Filtek supreme, Filtek supreme flowable, Filtek Bulk, Filtek one bulk fiill) | Vicker’s surface microhardness test | Polished specimens soaked in ethanol/ water or water for 1h, 1d and 30d then Vicker's (300g, 15 sec) hardness test. | Hardness increased after 24h (except Ever X), decreased after 30 days in water. Filler content related to hardness. Hydrolytic distribution of matrix and fibres cause decrease SFRC hardness. SFRC absorb more water than PFC. | Material, solvent and time effect hardness. Hardness influenced by matrix structure. SFRC should be covered with PFC to reduce degradation when exposed to fluids. CR composition and solvent both effect the surface of CR. |
| Oja et al. (2021)^18^ | Effect of accelerated aging on some mechanical properties and wear of different commercial dental resin composites | PFC - Supreme XTE, G-aenial, Denfil Bulk -Filtek Bulk fill, SFRC - Ever X posterior. | Vicker’s surface microhardness test | Vicker’s hardness with 1.96N, 10 sec and x40 objective review on the top surface only | Hydrothermal accelerated ageing had no significant effect on hardness and of CR including SFRC. Expired CR decreased surface hardness. | Hydrothermal accelerated ageing has no effect on microhardness. Filler particles have large influence on microhardness. Surface layer of unpolymerized resin found in the oxygen inhibition layer decreases microhardness |
|  |  |  | Two-body wear | Chewing simulator for two body wear was used with water. Wear facets were analysed with 3D optical microscope. | Hydrothermal accelerated ageing has no significant effect on wear depth of CR including SFRC. Expired CR did not affect wear of CR. | Many different physical and mechanical properties need to be examined to deem composite clinically useable. |
|  |  |  | SEM | SEM, two fractured specimens from each CR selected to be evaluated. | Conventional structure present on all CR. Small pits found from particle exfoliation. |  |
| Kumari et al. (2019)^19^ | Evaluation of surface roughness and hardness of newer nanoposterior composite resins after immersion in Food-Simulating Liquids | Surefil SDR, Clear fill majestic posterior, Ever X posterior, Tetric Evoceram, Filtek Z350 Minimal OIL, flash and air bubbles, samples were subject to air, water, ethanol or citric acid | Surface roughness | Contact stylus to measure surface roughness (2µm diameter), Ra value recorded | Surface roughness was material dependent; Clearfil, Filtek, Ever X, tetric then SDR. | Surface roughness is linked to hydrolysis. SFRC have less change. Roughness is affected by fillers and matrices. Roughness is increased when stress is placed on the fibre-silane-matrix bond. |
|  |  |  | Vicker’s surface hardness test | Vickers microhardness measurement with 15sec dwell at 500g force | Hardness was material dependent; clearfil, filtek, ever X, tetric then SDR. Ever X was not affected by any of the liquids. | Surface hardness and surface roughness appear to be affected by similar factors such as resin matrix and filler particles. |
|  |  |  | SEM | Observation at x1000 | Significant differences with material type and liquid type. | Different chemical compositions effect surface characteristics. Ethanol releases more monomers from CR. |
| Garoushi et al. (2015)^20^ | Influence of increment thickness on light transmission, degree of conversion and micro hardness of bulk fill composites | Bulk fill:X-tra fil, Venus bulk fill, Teric EvoCeram Bulk fill, SDR, Filtek Bulk fill and Sonic Fill. Conventional: Filtek superme, G-aenial anterior and Alert Flowable: G-aenial universal flow SFRC: Ever X Posterior | Vicker’s surface microhardness test | Samples were cured with a mylar strip on top, a x40 objective lens with a load of 981.2 mN applied for 10 S | Depth of cure correlates to hardness and influenced by material and thickness. Light transmission lowest for Alert. Ever X posterior was comparable to bulk fill PFC at 4mm. Similar depth of cure and thickness for high light transmission. | Longer curing time and depth of cure is related to hardness. Depth of cure is related to degree of conversion. Top bottom percentage must be at 80%. All CR expect Alert, Tertic EvoCeram and G-aenial anterior can be used to 4mm. |
| Raju et al. (2021)^21^ | Dimensional stability of short fibre reinforced flowable dental composites | 3 flowable resins with various fibre lengths at 5%. A control (no fibres), B 250µm (50 aspect ratio), C 350µm (70 aspect ratio) | Vicker’s surface microhardness test | Samples were cured then left for 24h and tested with Vickers surface hardness at 0.5mm increments. | Hardness at 0mm was similar. At 0.5mm the no fibre sample declined by 10% and decreased at 5mm. Fibre CR had a similar decline until 8mm. When it dropped below 80% required for clinical use. | Resin Composites decrease in hardness incrementally. SFRC may have up to 8mm depth of cure to clinically acceptable range. |
|  |  |  | Water sorption and solubility | 15mm diameter at 1mm thick samples were used | No significantly different findings found between samples. However, with decreased degree of conversion increases water sorption and solubility. | The glass fibres allow light to pass through the sample allowing greater curing depth and intensity. This had a negligible effect on water sorption and solubility. |
| Abouelleil et al. (2015)^22^ | Comparison of mechanical properties of a new fiber reinforced composite and bulk filling composites | Bulk fill resins: X-tra base, Venus Bulk Fill, Filtek Bulk fill, Sure fil SDR, Teric evoceram bulk fill, Sonicfill. Ever X posterior (SFRC) | Vickers surface microhardness test | Vicker’s hardness test was used with 200g of for 30 sec at 2mm and 4mm. | Sonic fill, Ever X posterior, Tetric evoCeram, Xltra Base were significantly harder than Surefill, Filtek Bulk and Venus bulk. Ever X posterior, Terric evoCeram and Venus Bulk 2 and 4mm were not different but others were softer at 4mm. | Ever X posterior can be used up to 4mm of cure without detrimental clinical failure. Short fibre reinforced composites have comparable or higher hardness to other bulk filled PFC resins. |
|  |  |  | SEM observation | Scanning Electron Microscope used to examine the fracture mode and fibre dimensions | Fibres stop crack propagation. Fibres are well dispersed. Fibre dimensions of ever X posterior are 16µm diameter and 1-2mm long. | The results show that SFRC can be used in stress bearing areas. |
| Attik et al. (2022)^23^ | Comparison of physical and biological properties of a flowable fiber reinforced and bulk filling composites | Ever X Flow, Filtek Bulk Fill posterior, SDR Flow + | Vickers surface hardness test | Cured CR followed by 24h soak in distilled water. Vickers hardness with 9.8N for 20sec and 200g for 30sec. Testing completed on the polished surface of the samples | Statistical variation to show that Filtek Bulk then Ever X Flow then SDR Flow + were the hardest. | Mechanical properties of exposed Ever X posterior show beneficial properties that could improve long term CR restorations |
|  |  |  | SEM |  | Variation in fibre orientation, and filler size. | Fibre, filler and matrices effect SFRC surface. |
| Garoushi et al. (2008)^24^ | Depth of cure and surface microhardness of experimental short fiber-reinforced composite | Experimental composites were made with 3mm E-Glass fibres were compared to conventional composite resin (3M-ESPSE) | Vickers surface microhardness test | Samples were cured for different lengths of time (20,40,60 sec) and tested with Vickers hardness. 1.96N for 10sec and observed with x40 objective. | Curing time increases surface hardness of SFRC. Microhardness correlations to degree of conversion. Filler particles and polymer matrix affect hardness. All CR are clinically appropriate. Water storage decreased surface hardness. Increased curing of SFRC decreases hardness due to plastising effect on matrix. | Clinical trials are needed for the fibre resins to confirm water sorption, hardness and longevity of the restorations. SFRCs are showing clinically acceptable results. Water acts as a plasticiser in the matrix Curing time increases monomer conversion |
| Mangoush, et al (2020) | Microstructure and Surface Characteristics of Short-Fiber Reinforced CAD/CAM Composite Blocks | Printing materials; Temp print medium, Pro3dure GR-17. Direct; Ever X flow, Essentia Universal. Lab; Gradia Plus. CAD/CAM; Cerasmart 270 and experimental SFRC (UDMA and TEGDMA with short fibres 200-300um and 7um diameter and fillers). | Two body wear | Localized wear tested then specimens placed in water for one day. 2 body wear simulator used (horizontal and vertical movements). At 2 kg equivalent to 15 000 loading cycles (1.5Hz and 20N chewing force) then measured with 3D non-contact opical profilometer (max wear recorded). | Lowest wear Temp Print, Cerasmart, Essentia Universal, Ever X flow and SFRC experimental, GR 17, Gradia Plus. Wear increased roughness on all materials. | SFRC resin CAD CAM blocks have promising behaviour for dental restorations. Particle size affects wear that affects other surface properties such as gloss and roughness. Short fibres used (200-300um) appear to have limited effect on the surface properties. Al products except of GR 17 (a temporary material) had sufficient gloss values. SFRC resins have adequate wear attributed to the shock absorbing capacity of the matrix and filler relationship. No relationship between wear and hardness. Hardness is affected by filler load and curing efficiency  Surface roughness could be due to loss of filler particles and abrasion of the resin matrix. Fibres polished with resin matrix not pulled out. As surface roughness decreases surface gloss increases. However the extent may vary depending on composite. |
|  |  |  | Surface roughness | Recorded before and after wear 5 x 0 objective lens and 0.5 multiplier. Ra values recorded. | Increase after wear on all materials, |  |
|  |  |  | Surface micro hardness (Vickers) | 1.96N for 10s and x40 objective lens 3 indentations for each sample, | Lowest; GR 17, Temp print, Essentia Universal, Ever X flow, Gradia Plus, SFRC block, Cerasmart 270. |  |
| Meenakumari et al. (2018)^26^ | Evaluation of mechanical properties of newer nano posterior restorative resin composites: an i*n vitro* study | Surefil SDR, Clear fill majestic posterior, Ever X posterior, Tetric Evoceram, Filtek Z250 | Nano hardness indentation | Nano indentation of microhardness force range; ≤30 nN to 10N. | Hardest to softest; SDR, Tetric EvoCeram, Filtek, Ever X posterior, Clear fill majestic posterior. | Type of fillers effect hardness. Significant differences in new CR. Clinical trials are needed. |
| Fráter et al. (2023)^27^ | Evaluation of microhardness of short fiber-reinforced composites inside the root canal after different light curing methods – An *in vitro* study. | 1) - control 3x2mm PFC (Grandia posterior) cured from top of root canal, 2) 3x2mm PFC (grandia posterior) cured from optic device that could enter Root canal, 3) 3x2mm SFRC (Ever X flow BULK) first 2 increments cured through fibre post, 4) 3 x 2mm SFRC (Ever X flow BULK) cured from optic device that could enter Root canal, 5) 2x4mm SFRC (Ever X flow BULK) first increment cured through fibre post, 4) 2x4mm SFRC (Ever X flow BULK) cured from optic device that could enter Root canal. | Microhardness | Completed at various depths of cure at 0.000252 μm/mN and microhardness was calculated accordingly. | Fibre posts increase microhardness. No significant difference in SFRC groups at 1mm, but with SFRC and fibre post at 3mm harder surface was recorded, the depth only effected the PFC groups. Elastic modulus increases with hardness with SFRC and PFC. Scattering of SFRC increased Depth of cure. | No advantage of layering SFRC in Root canal. The need for a novel Light curing unit that allows the greater guided light intensity into the fibre optics would be beneficial. Bulk fill SFRC is more beneficial than PFC in Root Canals |
| Garoushi et al. (2024)^28^ | A comparative Evaluation of short fibre-reinforced Composites | Essentia Universal (control), Alert, Fibrafill Flow, Fibrafill dentin, ever X flow, ever X posterior | Two-body wear | Vertical and horizontal chewing simulator. Average wear calculated with 3D optical profilometer | Least wear; Ever X Flow, Essentia, Alert, Fibrafill flow, Ever X posterior, Fibrafill dentin. Most wear. | Adding micro fillers does not increase wear of SFRC, high Filler: matrix load can lead to elastic deformation of the resin. Aspect ratio and Critical fibre length effect he wear of SFRC resins. |
| Lassila et al. (2019)^29^ | Characterization of a new fiber reinforced flowable composite | Surefil, Filtek Bulk Fil Flowable, Tetric EvoFlow Bulk Fill, Estelite Bulk Fill Flow, GC SFRC (experimental) | Water Sorption | Stored in water for 36 days, and measured at various times throughout | Highest to lowest; Estelite, Filteck, Surefil, Experimental, Tetric | Water sorption affected by hydrophilicity and cross-linking structure not inorganic filler content. Contributing factors: porosity, filler properties and filler interface. |
|  |  |  | Two-body wear | Polished samples, one day of water storage then two body wear test and chewing simulator. Observed with a 3D optical microscope. | Highest to lowest; Estelite, Filtek, Surefil, Tetric, SFRC. | Short fibres did not worsen wear of SFRC. Short fibres were also worn down with the resin matrix. |
|  |  |  | Microscopic Analysis | SEM Microstructure, fractographic and wear examination. Samples were covered with gold then observed. | SFRC SEM analysis showed orientation of random fibres and good wettability. Fractured fibres polished with the matrix. | Random orientation of fibres can cause plucking but no effect on physical characteristics of CR. |
| Lassila et al. (2020)^30^ | Characterization of restorative short-fiber reinforced dental composites | Alert (control), NovaPro Flow, NovaPro Fill, Ever X Flow, Ever X posterior | Two body wear | Polished composite in a chewing simulator with water with. Wear observed with 3D optical profilometer after. | Lowest to highest: Ever X flow, NovaPro Flow, Alert, NovaPro Fil, Ever X posterior | Microfibre in SFRC does not worsen the wear. No need for PFC surface coverage. But water sorption can cause an increase in monomer instability that can lead to greater wear susceptibility. Aspect ratio and critical fibre length can help predict wear fo SFRC resins |
|  |  |  | Water sorption | Samples soaked in water for 36 days measured periodically throughout | Lowest to highest: Alert, NovaPro Fil, Ever X Flow, Ever X posterior, NovaPro Flow. | Low filler content in SFRC increases water sorption. Hydrophilicity, polymer matrix and chemical stability effect water sorption. |
|  |  |  | Microscopic analysis | After one day of soaking in water samples were analysed by gold covering then SEM | Fibre diameter effect mechanical characteristics of CR. | Various fibre aspect ratios affect physical characteristics of CR. |
| Garoushi et al. (2017)^31^ | Mechanical Properties and Wear of Five Commercial Fibre Reinforced Filling Materials | Alert, Easy Core, Build-it, TI-Core, Ever X posterior, Enamel (Control) | Surface wear – Chewing simulator and two-body wear test | Mylar strips used to help cure then polished. 1d water storage then 2 body wear in chewing simulator, vertical and horizontal movements in water. Followed by 3D profilometer microscope. | Least wear to most; Enamel Easy-Core, Build-it, Alert, ever X posterior, TI -Core. SFRC surface properties are dependent on fibre dimensions, fibre orientation, fibre loading and fibre matrix adhesion. | Good wear to Easy Core compared to Alert and ever X due to filler particles not fibre strength. SFRC are recommended to be covered with PFC except Alert. |
|  |  |  | SEM | Provided characterisation of SFRC. Gold spluttered before SEM. | Different filler particle size, shape and orientation give reason for the physical characteristics | Microstructure affects properties of wear. Air bubbles affect polishability of CR. |
| Asadian et al. (2021)^32^ | Evaluation of Wear Properties of Four Bulk-Fill Composites: Attrition, Erosion, and Abrasion | Ever X posterior, Filtek Bulk- posterior, Sonic Fill 2, Xtra Fill, Filtek Z250 Universal (control) Weight loss and surface roughness measured. | Attrition wear (chewing simulator) | Chewing simulator, sound human teeth are used as antagonists. 50N of force with 0.8mm lateral excursion with 250,000cycles. | Xtra composite had significantly more loss than ever X and Filtek. Ever X and Filtek were not different in weight loss. No surface roughness difference. Surface roughness decreased in all specimens but not significantly. Clinical acceptance was kept in all CR except for x-tra fil. Increases in Ra relates to particle filler. | Presence of matrix TEGDMA effects wear rate and surface roughness. Filer shape affects wear rate too. Fibres distribute stress better. Clinical studies are needed to allow for more accurate findings. Wear can be affected by the filler particles present and thus the wear rate of the matrix and filler particles can be different causing increased roughness. |
|  |  |  | Erosion Wear (pH cycling) | pH cycling for 5 days, demineralized for 6h (4.7pH) then remineralized for 18h (pH 7). |  |  |
|  |  |  | Abrasion wear (tooth brushing) | Mechanical tooth brushing with toothpaste and water. 100 000 cycles. |  |  |
| Sarthaj et al. (2018)^33^ | Comparative evaluation of wear resistance of cast gold with bulk-fill composites an *in vitro* study | Type IV casting alloy (gold alloy), Ever X posterior (SFRC), Teric N-Ceram Bulk fill (nanohybrid resin Composite) | Pin-on-disc wear test | Pin-on-a-disc wear tester used to test wear at 23^o^C for 2 minutes at 50 m/s. The difference in weight was measured. | Highest wear to lowest: Teric N-Ceram Bulk fil, Ever X posterior, Type IV alloy | Bulk SFRC have higher filler content and lower wear compared to PFC resins. Clinical studies are needed. For fibres to be successful to reduce wear they must exceed the critical fibre length. |
| Callaghan et al (2006)^34^ | Effect of fiber volume fraction and length on the wear characteristics of glass fiber-reinforced dental composites | Composite resins with varying fibre lengths and percentage of filler; 3-2.0% 1.5mm, 3-5.1% 1.5mm, 3-5.7% 3mm, 3-7.6 1.5mm against 5.1% 3mm fibres | Pin-on-disc wear test | Pin on a disc with no water present, samples are in half spherical shape, tests were conducted at 60rpm. | 2.0% and 7.6% had higher wear. 2.0% (adhesion and abrasion), 7.6% (fibre plucking from clustering). PFC has superior wear compared to FRC, but 5.7% has better wear after a critical point. Microcracks formed in the PFC. | Fibre content and matrix ratio is not linear to wear. Microcracks accelerate wear and found more on PFC, but less <4.45N. transition period of FRC is not corelated to fibre length. 5.7% has superior wear resistance. Fibre plucking can cause increased wear and is increased if fibre bundling occurs. |
| Lassila et al. (2024)^35^ | Bacterial adhesion and surface roughness of particulate filled and short fiber-reinforced composites | Cream X Universal, Filtek Universal, Ommi Chroma, Tetric Prime, Venus Diamond, Ever X Posterior, Ever X Flow Dentin, Ever X Flow BULK, Experimental SFRC (Short fibre 78%) | Surface roughness | Non-contact optical profilometer assessed before adherence tests, Ra value calculated in average line across specimen. | Lowest Ra value: Ever X Flow dentin and BULK, the highest Ra Value: Ever X posterior and Cream X Universal. Fibres were visible in Ever X Posterior but not ever X Flow. | Weak relation between roughness and bacterial adhesion. Particle/ filler proportion and size, particle/ fibre exposure and air bubbles effect surface roughness. All CR are clinically acceptable. Resin matrices may also affect bacterial adherence through their ability to adjust the surface properties, namely the surface energy present. |
|  |  |  | Bacterial Adherence | Adherence of *S. mutans* | Bacterial adherence has been thought to be related with surface energy however SFRC have increased surface energy compared to PFC but similar bacterial adherence. |  |
|  |  |  | SEM analysis | 5min fixation, then rinsed with water and dried with ethanol for 10 min. SEM observation of samples. | Filler/Fibre diameters affect surface roughness measurements. | Uniform distribution of bacteria on fibres, fillers and matrix. No favouritism to surface energy. |
| Lassila et al. (2009)^36^ | Adherence of *Streptococcus mutans* to Fiber-Reinforced Filling Composite and Conventional Restorative Materials | Filtek Z250, Grandio, Nulite, FUJI II LC, Amalgam, ever Stick, Cerena class I, Experimental SFRC (22.5wt% of short 2-3mm Glass E fibres and 22.5wt% of resin matrix and 55wt% filler particles) Enamel, Dentin | Surface roughness | Surface profilometer with cut off at 0.8mm, transverse stylus speed of 0.1mm/s | Surface roughness and bacterial adhesion are not related. The Fibre reinforced composites had the highest roughness values to other composites. Surface preparation can cause air bubbles that can affect roughness. | SFRC surface roughness of SFRC was the highest but still clinically accepted. Matrix, ratio and size of fillers affect roughness. Fillers relate to polishablity of CR. Surface roughness as a Ra value is within the clinically accepted range. |
|  |  |  | Bacterial Adherence | Surface free energy is a related factor to bacterial adherence (*S. Mutans)* | Increased surface free energy can be reported with SFRC but this does not translate to bacterial adhesion. | The presence of fibres on the surface of SFRC has cause increased surface energy but this has not translated to increased bacterial adhesion. The bacterial load is with in clinically accepted range for SFRC. |
|  |  |  | SEM Analysis | Samples fixed for 5min, rinsed with water, dried with ethanol, and SEM observation. | Less bacterial adherence after saliva coating. | The SEM unable to detect the attachment of *S. Mutans* due to uneven surfaces. |
| Jafarnia et al. (2021)^37^ | Physical and mechanical characteristics of short fiber-reinforced resin composite in comparison with bulk-fill composites | SFRC (ever X posterior), BULK (beautiful Bulk and Filtek Bulk) | Surface roughness | Samples cured and polished. Ra determined with profilometer at 0.8mm and 0.1mm/sec. Roughness examined before and after different grit sandpapers (non-polished, #80, #400) | Roughness of Ever X increased with all polishing. #80 grit increased all specimens, #400 polished PFC, by reducing roughness. | Filler size and shape effect polishability along with sandpaper grit. Ever X is a promising base material composite |
| Łagan et al. (2023)^38^ | Surface free energy and roughness of flowable dental composites | Ever X Flow BULK, Ever X flow dentin, Flow art. Control was after polymerization but before sandblasting | Roughness - 3D optical profilometer (ra, Rz and sa,sz measured) | A is the arithmetic mean whilst z is the maximum roughness. | Sandblasting increases roughness to all CR biggest difference was with ever X flow dentin. | Sandblasting increased the roughness of both flowable, and bulk filled SFRC however this was more consistent with Bulk filled SFRC resins. |
|  |  |  | Contact angles | Diiodomethane and a-bromonaphthalene, glycerol, distilled water | Wettability of ever X bulk and flow are similar. ever X dentin has less wettability | Contact angle and surface free energy factors effect adhesion and bonding. Initial state of surface was hydrophilic, but roughing caused hydrophobic characteristics. Sandblasting roughness did not affect free energy. |
|  |  |  | Surface free energy | the Owens–Wendt’s and van Oss Chaudhury–Good’s methods were used to obtain critical surface tension | Sandblasting decreased Flow art surface free energy. Ever X composites did not change. |  |
|  |  |  | Critical surface energy | Zisman’s method | Increase in all surfaces after sandblasting. | Tissue bio-adhesion is affected by critical surface energy and sound for all tested CR. |
| van Dijken et al. (2006)^39^ | Fiber-reinforced packable resin composites in Class II cavities | Nulite F with NS universal bond adhesive, Alert (Amalgam Like Esthetic Restorative Treatment) with Bond-1 on medium to large Class II cavities in a clinical scenario of Amalgam replacement. | Clinical Surface roughness | Modified USPHS criteria blind evaluation –Surface roughness evaluated at 6m, 12m, 2y, 3y, 4y, 5y, and 6y | 14 restorations (9 Nulite, 5 Alert) failed in 6 years 0 surface properties. Nulite roughness increased (1 and 2 years) then decreased (year 3). Fibres visible microscopically in both materials. Loss of fibres and increased roughness at 6 years with clinical matrix wear and fibre loss. | Matrix monomers, filler particles, fibre geometry, fibre amount, fibre size, and adhesive system affect CR surfaces. Alert had failure rate higher than clinical acceptance Nultie did not. Patient selection may cause high failure rate. Most failures were after 3 years so long-term evaluation of CR are needed. Surface roughness may be an associated factor but was not deemed to be the primary reason for failure of restorations. |
| v NovaPro. (n.d.)^40^ | Strength and Aesthetics in One Composite | Nanova pro | Water sorption | Un stated | Novapro Fill about 10ug/mm^3^ Filtek supreme about 32ug/mm^3^ | Water weakens composite and lessens the lifespan. |
| Alshabib et al. (2023)^41^ | Effect of Fibres on Physico-Mechanical Properties of Bulk Fill Resin Composites | Ever X flow, Activa, Palfique Bulk flow, Filtek Bulk flow, SDR flow composite | Water sorption and Solubility | Samples cured and placed in water and measured at 1, 7, 14, 21, 28 and 60 days. | All composites were within the ISO standard for water sorption and solubility. Water diffusion occurs through resin matrix. Capillary action of fibres increase mass. | It is advisable to cap dentin replacement composites (Ever X, SDR flow) with PFC or place away from high occlusal load. Water sorption is the gaining of water within a material. Solubility is the ability for a material to become soluble in water |
